# Identifying and training deep learning neural networks on biomedical-related datasets

**DOI:** 10.1093/bib/bbae232

**Published:** 2024-07-23

**Authors:** Alan E Woessner, Usman Anjum, Hadi Salman, Jacob Lear, Jeffrey T Turner, Ross Campbell, Laura Beaudry, Justin Zhan, Lawrence E Cornett, Susan Gauch, Kyle P Quinn

**Affiliations:** Arkansas Integrative Metabolic Research Center, University of Arkansas, Fayetteville, AR; Department of Biomedical Engineering, University of Arkansas, Fayetteville, AR; Arkansas Integrative Metabolic Research Center, University of Arkansas, Fayetteville, AR; Department of Computer Science, University of Cincinnati, Cincinnati, OH; Department of Computer Science and Computer Engineering, University of Arkansas, Fayetteville, AR; Arkansas Integrative Metabolic Research Center, University of Arkansas, Fayetteville, AR; Department of Computer Science and Computer Engineering, University of Arkansas, Fayetteville, AR; Department of Computer Science and Computer Engineering, University of Arkansas, Fayetteville, AR; Health Data and AI, Deloitte Consulting LLP, Arlington VA, USA; Health Data and AI, Deloitte Consulting LLP, Arlington VA, USA; Google Cloud, Reston VA, USA; Arkansas Integrative Metabolic Research Center, University of Arkansas, Fayetteville, AR; Department of Computer Science, University of Cincinnati, Cincinnati, OH; Department of Computer Science and Computer Engineering, University of Arkansas, Fayetteville, AR; Department of Physiology and Cell Biology, University of Arkansas for Medical Sciences, Little Rock, AR; Department of Computer Science and Computer Engineering, University of Arkansas, Fayetteville, AR; Arkansas Integrative Metabolic Research Center, University of Arkansas, Fayetteville, AR; Department of Biomedical Engineering, University of Arkansas, Fayetteville, AR

**Keywords:** cloud-based computing, deep learning, artificial intelligence, biomedical research, engineering education

## Abstract

This manuscript describes the development of a resources module that is part of a learning platform named ‘NIGMS Sandbox for Cloud-based Learning’ https://github.com/NIGMS/NIGMS-Sandbox. The overall genesis of the Sandbox is described in the editorial NIGMS Sandbox at the beginning of this Supplement. This module delivers learning materials on implementing deep learning algorithms for biomedical image data in an interactive format that uses appropriate cloud resources for data access and analyses. Biomedical-related datasets are widely used in both research and clinical settings, but the ability for professionally trained clinicians and researchers to interpret datasets becomes difficult as the size and breadth of these datasets increases. Artificial intelligence, and specifically deep learning neural networks, have recently become an important tool in novel biomedical research. However, use is limited due to their computational requirements and confusion regarding different neural network architectures. The goal of this learning module is to introduce types of deep learning neural networks and cover practices that are commonly used in biomedical research. This module is subdivided into four submodules that cover classification, augmentation, segmentation and regression. Each complementary submodule was written on the Google Cloud Platform and contains detailed code and explanations, as well as quizzes and challenges to facilitate user training. Overall, the goal of this learning module is to enable users to identify and integrate the correct type of neural network with their data while highlighting the ease-of-use of cloud computing for implementing neural networks.

This manuscript describes the development of a resource module that is part of a learning platform named ``NIGMS Sandbox for Cloud-based Learning'' https://github.com/NIGMS/NIGMS-Sandbox. The overall genesis of the Sandbox is described in the editorial NIGMS Sandbox [1] at the beginning of this Supplement. This module delivers learning materials on the analysis of bulk and single-cell ATAC-seq data in an interactive format that uses appropriate cloud resources for data access and analyses.

## INTRODUCTION

Image processing and data analysis are important tools for all engineering and life science disciplines [2]. For biomedical-related engineering practices, data analysis provides the ability to identify biologically relevant patterns hidden within data that can be used to draw conclusions regarding everyday life [3, 4]. To understand complex biological pathways and relationships, the amount of data required grows exponentially, leading to multi-dimensional datasets that are difficult to understand. Additionally, as the quantity of data increases, datasets may contain relationships that are not well known or understood. This issue is further compounded by technological advancements in biomedical imaging, where improvements in spatial and temporal resolution can lead to large three-dimensional or four-dimensional image datasets. Although there are many well-established tools for multi-dimensional data analysis, the patterns that are identified may contain some type of systematic or user bias. Therefore, advanced data analysis tools are needed to objectively identify complex patterns hidden within data, which then can be utilized to draw meaningful conclusions.

Artificial intelligence (AI) broadly defines the simulation of human intelligence via computational models and equations. Machine learning refers to the use of models and dimensionality reduction to allow for a computer to ‘learn’ relationships within a dataset [4]. A neural network is a type of machine learning model that mathematically simulates the neuronal connections in the human brain to process and understand data [5]. The smallest base unit for a neural network is a perceptron, and a neural network is formed by connecting perceptrons with each other. The strength of a single connection is defined as a weight, and layers of perceptrons have an additional bias term. The weights and biases of a neural network are used to dictate the flow of data through a neural network, where the final output is used to incrementally ‘train’ a neural network to perform some task. These tasks typically fall into one of two categories: classification or regression. For classification tasks, the final network prediction is a discrete outcome for an entire input and falls into one of multiple user-defined classes. Segmentation, which is a type of classification task, also predicts discrete outcomes, but often on a pixel-by-pixel basis. Alternatively, regression tasks are used to predict continuous values. When identifying what type of network to use, it is important to consider the specific research question that is being asked, as well as the desired outcome. For example, in images containing tumor pathology, such as in the PathMNIST dataset [6], a classification network can be used to determine if an image contains a tumor. A segmentation network can be used to determine where the tumor is located in an image, and a regression network could be trained to estimate the size of the tumor. These neural networks begin as a model with randomized weights and biases that are trained by incremental learning patterns from a relatively large pool of data, also known as a training set. After each iteration of training (or epoch), the neural network accuracy is quantified from the training set and cross-checked with a smaller validation set to determine if the model is overfitting the training data. Once the network completes many epochs and training is completed, the patterns the network learned can be used to predict outcomes from new data, referred to as a testing set that the network has not previously seen.

Deep learning is a subset of machine learning where a ‘deep’ neural network, containing two or more interconnected layers of perceptrons, is modeled and trained [7]. The most basic form of a deep neural network is the multi-layered perceptron, which contains multiple hidden layers of perceptrons, where each layer is fully connected to the previous layer with a set of weights [4, 8]. For biomedical image datasets, however, the most common type of deep neural network used is a convolutional neural network (CNN) [3]. A CNN is a type of deep learning model that utilizes many small kernels that are adjusted to be sensitive to image features within the dataset through training. Within a convolution layer, kernels are scanned across the input to determine if the input contains those features. To make these types of networks ‘deep’, many of these convolution layers are used, and the feature maps produced by convolution layers are further highlighted by pooling layers, which isolate the most important features contained in the feature maps. Finally, the output from the last convolution layer is flattened into a vector and used to output a single classification for an input. In the biomedical application space, CNNs have previously been used for detecting cancer [5], segmenting cells and tissue [9, 10] and tracking particles [11] and has general uses in bioinformatics [4]. Deep learning neural networks are fast and can produce human-like accuracy without user input. However, one of the major downsides limiting the broader use of deep learning neural networks in biomedical research is the learning curve associated with these tools. Specific hurdles include: identifying the correct type of neural network to use, determining how to create the network in a programming language, obtaining computational resources sufficient to train the networks and knowing how to sufficiently train the network. Open-source deep learning platforms, such as TensorFlow or PyTorch, can allow users to quickly generate deep neural networks [12]. Additionally, these platforms contain many examples on how to get started with generating and training neural networks. However, these examples are primarily focused on large generic datasets that are meant to highlight the capabilities of deep learning neural networks but are far removed from biomedical research. The application of deep learning neural networks on biomedical datasets is severely lacking in publicly available training examples, resulting in confusion from users on how to adapt or use neural networks for biomedical research.

**Figure 1:**
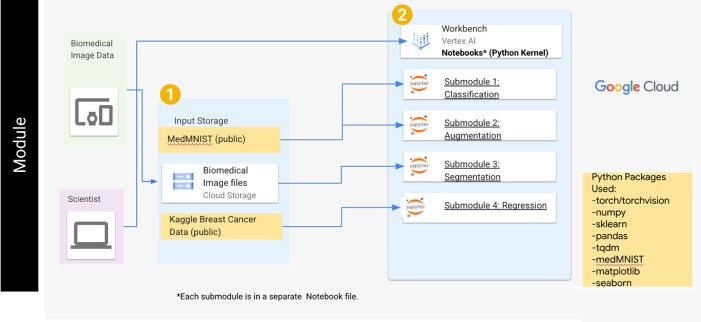
The general user workflow for the deep learning module. The user can access the submodules sequentially through the Vertex AI workbench and will use multiple datasets within each submodule. For the classification and augmentation submodules, datasets within the MedMNIST library are used. A cloud storage bucket containing an image dataset is used for the segmentation submodule, while a public dataset regarding breast cancer data is used in the regression submodule.

The goal of our learning module is to introduce new users to how deep-learning neural networks can be properly utilized for biomedical datasets. To achieve this goal, the module is divided into four submodules that cover image classification, data augmentation, image segmentation and data regression. Overall, each submodule is comprised of a single python-based Jupyter notebook for increased user readability and utilizes the PyTorch library for data preparation and neural network training (Figure 1). Within each submodule, the background and motivation for a specific technique, along with the steps needed to prepare a dataset for network training, generating a neural network, training a neural network and quantifying the network’s performance, are included. Key scientific concepts are also discussed within each submodule, and knowledge checks, exercises and challenges are included to further test the user’s understanding. Finally, these modules are freely available on GitHub and are set up to be used asynchronously on cloud-computing resources, which reduces the hardware requirements and allows for large dataset storage that is easily accessible to the end user. To support the generation of these modules, the Vertex AI product within the Google Cloud Platform (GCP) is used to enable quick setup of virtual machines. By generating this module, we have allowed for a means of introducing difficult deep learning concepts and increasing the ability for users to properly utilize neural networks on biomedical-related datasets. This article describes the algorithms used in each submodule, their implementation within the GCP, typical results obtained when users run each submodule and discussion of how these submodules can aid new biomedical researchers interested in utilizing deep learning CNNs.

## METHODS AND IMPLEMENTATION

### Submodule 1: classification

Classification refers to the process of determining some discrete outcome based on continuous features within input data. Although this practice is commonly used by researchers and clinicians, training and utilizing professionals to accurately classify relatively large (>100 000 samples) datasets is costly and time-consuming. Recently, CNNs have been growing in popularity and have been shown to complete a multitude of different tasks [7, 8]. When completing a classification task using deep learning, the first step is to properly identify what type of architecture to use. Experimenters can develop their own custom architectures to suit their needs, but this technique may be difficult depending on previous experience. An easier initial approach is to use a pre-defined CNN architecture. There exist many ‘gold standard’ pre-defined architectures, such as ResNet-18 [13], AlexNet [14] or VGG [15], as well as many more [7]. Each pre-defined CNN architecture has different advantages such as implementation of novel convolution block types or convolution block connections (i.e. skip connections). Moreover, these pre-defined architectures are widely available as either untrained or pretrained on a relatively large dataset such as ImageNet [16]. Utilizing predefined CNN architectures are a good starting point with a new dataset and provide a quick means of getting started with deep learning. One common practice when initially using predefined CNNs is to use a technique called transfer learning, where a pre-trained network is fine-tuned based on a new dataset. This process involves freezing the weights and biases within the network and re-training the last few layers based on the number of classes within the new dataset [17]. While any number of pre-defined architectures can be used, the ResNet-18 network is used in this submodule to demonstrate the concepts and capabilities of a CNN and transfer learning.

**Figure 2:**
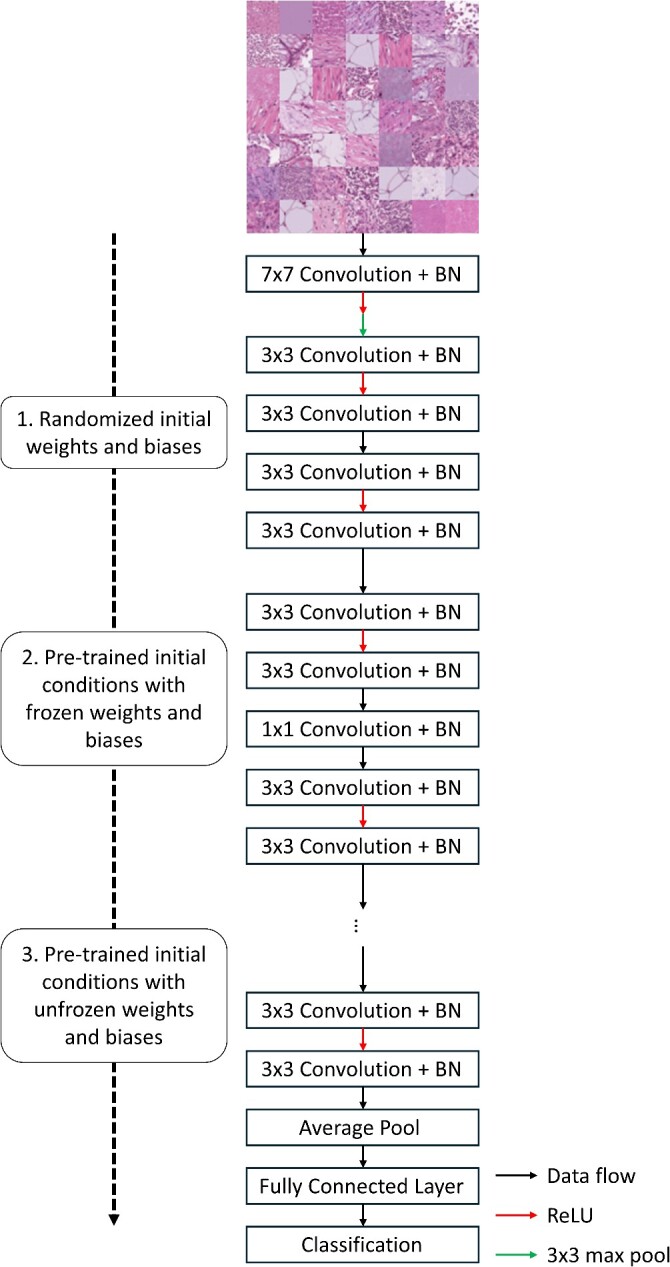
Graphical overview for the classification submodule. The PathMNIST dataset is used to train a ResNet-18 network based on different initial conditions: randomized initial weights and biases (first experiment), pre-trained network weights and biases that are frozen during training (second experiment) and pre-trained network weights and biases that are unfrozen during training (third experiment). Following network training, the prediction accuracy of the three networks is compared.

This submodule is divided into three ‘experiments’ that use the ResNet-18 network architecture and three levels of transfer learning to highlight common methods used when training CNNs (Figure 2). The ResNet-18 architecture is comprised of a convolution operation, followed by a max pooling operation, and then five residual blocks, each with two convolution operations. Finally, an average pooling operation is used to vectorize the final output, and a softmax operation is used to predict the class of an input image. For each experiment, the network is trained on the PathMNIST dataset, which is a dataset containing RGB images (107 180 images total) of colon pathology used to predict cancer prognoses based only on stained images [6, 18]. Prior to the three experiments, the PathMNIST dataset is loaded, and the images are normalized to have a mean pixel intensity of 0.5, as well as an intensity standard deviation of 0.5. The images are then randomly sorted into a training, validation and testing dataset. To maintain consistency across each experiment, each network is trained using a stochastic gradient descent with momentum (SGDM) optimizer with the default training parameters (learning rate = 0.001, momentum = 0.9). Since these experiments are for only comparing different methods of network training and not for producing high-quality classification results, each network is trained for five epochs. Finally, the loss for each network is calculated using the cross-entropy loss algorithm [19]. Following the network training process, the overall multi-class accuracy is calculated as the ratio of correctly predicted training images to the total number of training images. The accuracy of each model is then compared to determine the best model for this application.

For the first experiment of this submodule, a neural network is created based on the ResNet-18 architecture with random weights and biases and then trained on the PathMNIST dataset. The second experiment consists of using a pre-trained version of the same network architecture. With this experiment, the weights and biases within the network are frozen to prevent re-training. The final classification layer is then removed from the pre-trained network and replaced with a new classification layer with the correct number of output classes. For this experiment, only the last classification layer is re-trained on the new dataset. Finally, the third experiment uses the pre-trained model and continues to use all the weights and biases within the network on the new dataset [17].

### Submodule 2: augmentation

**Figure 3:**
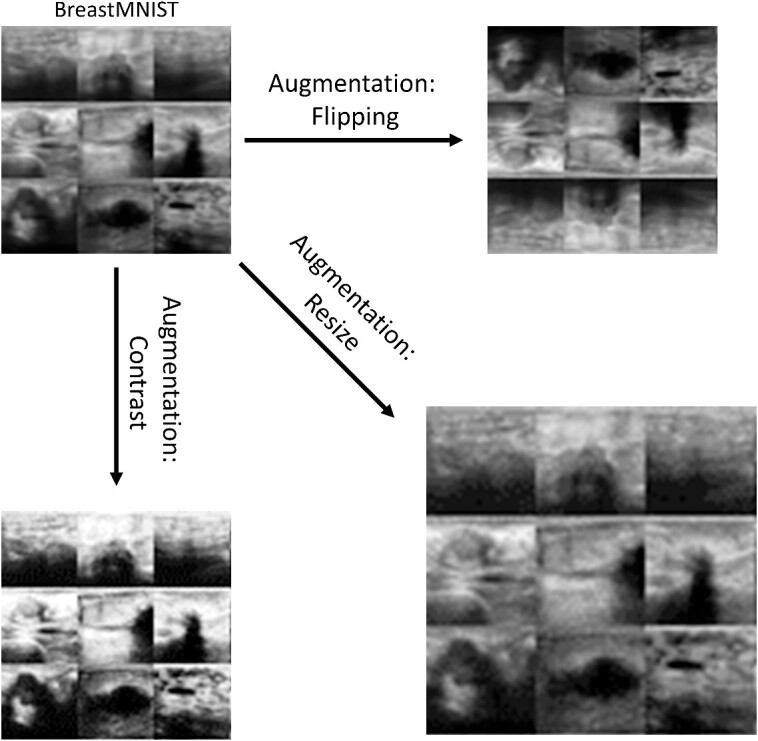
Examples of data augmentation using the BreastMNIST dataset. The types of augmentation shown are horizontal and vertical flipping (top right), scaling (bottom right) and contrast adjustment (bottom left).

Although CNNs are powerful and relatively accurate tools for detecting key features within data, the collected data may contain some inherent biases that may cause the network to incorrectly learn patterns associated with human preferences in data collection. Additionally, during the training process of a neural network, the same training set is recycled many times to incrementally train the network. This may result in overfitting the training dataset (particularly when the dataset is small), which will result in poor generalization for the network [7]. To mitigate these issues, a dataset can be transformed or augmented. Augmentation refers to the process of synthetically creating more image data through transformations such as rotation, scaling and cropping [7, 20]. An augmented dataset contains a random combination of normal and augmented data, which is then used to train the network. In this submodule, different types of data augmentation are discussed and visualized. Additionally, the ResNet-18 network architecture is trained on a dataset containing no augmentation and its accuracy is compared to a network trained with random cropping and flipping of the dataset. Finally, a network is trained on a dataset that combines the normal and augmented dataset (Figure 3).

In this submodule, data augmentation is explored through data visualization and quantifying the effect it has when training a CNN. To visualize different augmentation methods, the ChestMNIST dataset is used, which contains grayscale images of chest x-rays (112 120 images total) that contain images of eight different common thoracic diseases [18, 21]. For training the neural networks, the BreastMNIST dataset, which contains 780 images of cancerous and non-cancerous breast ultrasounds, is used [18, 22]. Although there are many different augmentation methods and algorithms that are commonly used, datasets within PyTorch can be easily augmented using the ‘torchvision’ package. Using this package, this submodule visualizes images that are augmented via random horizontal flipping, rotation, resizing and cropping, as well as changes in brightness, contrast, saturation and hue.

When performing augmentation on a dataset, it is important to identify the types of augmentation that would result in improved accuracy. In this submodule, the same CNN is trained using unmodified data, only augmented data and a combination of the two. The types of augmentation used in these examples consists of a combination of random horizontal flipping and random cropping. The effect of data augmentation on the prediction accuracy of a network is quantified by training ResNet-18 CNNs on either an unmodified dataset, a completely augmented dataset or a combined dataset containing both augmented and unmodified data. Similar to the previous submodule, each network was trained using an SGDM optimizer with cross-entropy loss as the loss criterion. Since the size of the augmented dataset is proportionally larger compared to the original dataset, a slightly higher training time of 10 epochs was used, and the final prediction accuracy was quantified as the ratio of correctly classified images to the total number of input images and compared among the three training conditions.

### Submodule 3: segmentation

As seen in previous submodules, CNNs can be trained to output a single classification based on a collection of features within input data. However, image data may have multiple regions of interest pertaining to different classifications (e.g. tumor versus healthy tissue), and it may be desirable to spatially resolve these regions of interest. One method for overcoming this issue is to train a classification CNN on a relatively small image input size and then allow the trained CNN to classify small sections of a larger input image. This will result in a classification map that may contain multiple class labels for a larger input, which is a process known as segmentation [23]. However, this process is time consuming and computationally inefficient. A more efficient approach is to train a fully CNN to predict some outcome at every location in the input image. For classification tasks, output prediction maps can also be fine-tuned to either only output the specific class on a per-pixel basis (i.e. semantic segmentation), or to predict the location of individual objects of interest (i.e. instance segmentation). For example, for PathMNIST images of colon tumors, semantic segmentation networks could identify pixel locations within the image containing tumor cells, while instance segmentation could identify the locations of tumor cells and count them.

**Figure 4:**
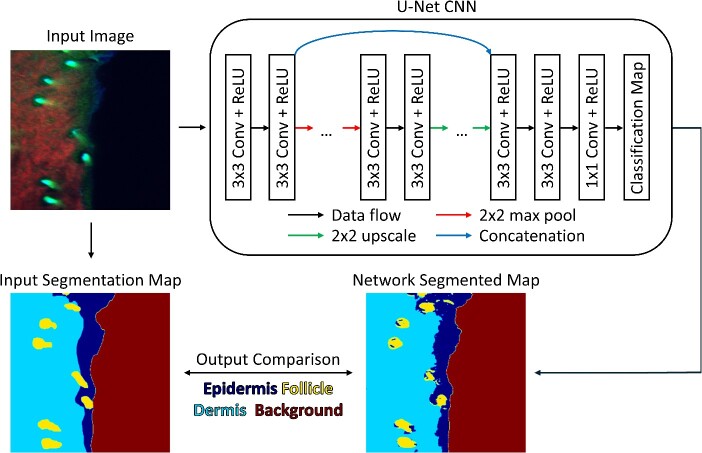
Graphical overview of segmentation submodule. Using a biomedical dataset of fluorescence images, a U-Net CNN architecture is trained to produce a semantic segmentation map based on four user-defined classes. During network training, the classification map produced by the neural network is compared to a user-defined segmentation map.

Semantic segmentation is an ideal method for identifying with high spatial precision where a specific user-defined class can be identified in an image. One of the first broadly successful architectures to implement this prediction scheme is the U-Net architecture [24]. This network contains both an encoding and decoding path for identifying simple and complex features within an input. This network then produces a classification map that is the same size as the original input. While the U-Net architecture is well suited for semantic segmentation, more advanced network architectures, such as YOLOR [25] or SAM [26], which combine groups of neural networks with complex intra-network relationships, have been used for efficient instance segmentation. In this submodule, the U-Net network is explored and applied to a biomedical image dataset for the purpose of delineating skin features (Figure 4).

The goal of the submodule is to describe the U-Net architecture and highlight its ability to segment input images. The first step in this module is to describe and generate the U-Net architecture, which is made up of four down-sampling blocks and four up-sampling blocks. To down-sample, or encode, the input image, two convolution operations are followed by a max pooling operation. Once data are encoded, they are up-sampled, or decoded, back to the original input image size by performing two convolution operations and a bilinear up-sampling operation. By using the ‘imageio’ package in python [27], an example image of tissue is loaded and passed through the initialized network and the output is observed. For this submodule, the dataset used is a subset of a previously published dataset containing RGB images (731 images total) of *in vivo* skin autofluorescence that were acquired using a multiphoton microscope [9, 28]. The goal of this dataset is to use image features to detect whether pixels are classified as epidermis, dermis, hair or background. Along with the input images, a set of corresponding labeled images are provided where each pixel is labeled based on a specific class [9, 28]. To enable network training on this dataset, a custom dataset class is generated, which loads an image and its labeled map pair and performs random horizontal and vertical flipping. For the network to be trained efficiently, all the image pairs are loaded into memory and each pair is randomly sorted into a training, validation or testing dataset using a 70%, 20% and 10% split, respectively.

When training a U-Net architecture, slightly higher training times must be used due to the number of trainable parameters within the network. However, since the dataset used in this submodule is substantially smaller relative to other submodules, a total of 10 training epochs are used and the amount of overfitting is carefully observed via the validation set. During training, a pixel-wise cross-entropy algorithm is used to calculate the loss. and an adaptive moment (ADAM) optimizer [29] with default settings (learning rate = 0.001) is used. To visualize the accuracy of the network during the training process, the average loss and accuracy of the training and validation datasets are plotted at the end of each epoch. Once the network has been trained, a ratio of the correctly classified pixels relative to the total number of pixels in the training, validation and testing datasets is calculated to quantify the network accuracy. Finally, the input image, ground truth map and predicted map from the trained network are displayed to allow the user to check the ability for the network to predict each class within an input image.

### Submodule 4: regression

**Figure 5:**
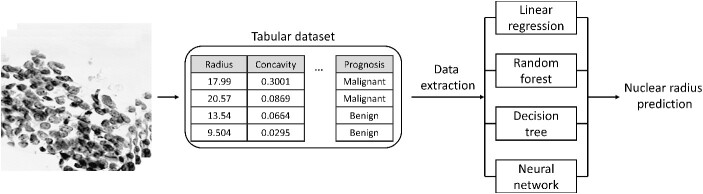
Graphical overview of regression submodule. An image-based tabular dataset containing continuous values describing the tumorous cells within images is typically used to predict a binary prognosis (malignant/benign). Using multiple approaches, the data within the tabular dataset is used to predict the continuous nuclear radius.

As shown in the previous submodules, a classification CNN is a tool that allows for simple and complex patterns to be selectively picked out and used to classify the input. However, the CNN output of a class for a given pixel or image is discrete, which may not be ideal when the desired output is actually a continuous number [13]. Regression refers to the method of training a neural network to predict a continuous value, rather than a discrete label. Due to the network output being continuous rather than discrete, there are important considerations when training these types of CNNs. In this submodule, the process of training a CNN to predict continuous values is explored (Figure 5).

For regression neural networks, features are extracted from an input and used to predict some continuous value. In this submodule, a tabular dataset (569 total entries) that characterizes cell nuclei in breast cancer masses by quantifying image properties such as radius, perimeter and area, is used [30, 31]. This dataset is first loaded into memory and visualized. Next, the measure of nuclei radius is identified as the target output for the regression model. When training a neural network with a regression output, the equations used to quantify network error are different from classification CNNs. Metrics that are commonly used include mean absolute error (MAE), mean squared error (MSE) and root mean squared error (RMSE). Although the PyTorch library allows for data manipulation and neural network training, there are many other Python-based packages that have regression models built-in, such as the popular ‘scikit-learn’ package [32]. To assess the accuracy of a CNN, the accuracy is computed using a simple linear model, decision tree and random forest regression model. Finally, a simple linear regression neural network is trained on the same dataset. Since regression networks are trained to output continuous variables rather than discrete classes, the amount of training time needs to be increased substantially. The neural network training phase consists of 100 epochs, and the accuracy of the network is assessed by calculating the MSE between the network-predicted value and the tabular value. To adjust the weights and biases within the network, a stochastic gradient descent (SGD) optimizer is used with a default learning rate of 0.0001. To compare what the neural network learned versus the other simple regression models, the most important features for each type of model are extracted and compared.

## RESULTS

### Submodule 1: classification

Fine tuning a pre-trained CNN to predict a discrete outcome is a useful method for determining the effectiveness of deep learning on a particular dataset. In this submodule, the ResNet-18 architecture is used to predict tissue classes based on an input image. When the network architecture is randomly initialized and trained on this dataset, a relatively high accuracy (≥90%) is achieved. Pre-defined network architectures are also available as pre-trained versions, which are typically trained on very large and diverse datasets. When using a ResNet-18 model that is pre-trained on the ImageNet dataset (>14 million images) [16], and only the final prediction layer is allowed to train, the accuracy decreases by about 20%. However, if the whole network is allowed to re-train, the accuracy is consistent with the randomly initialized network.

### Submodule 2: augmentation

Data augmentation is a common practice for improving the robustness of CNNs. By randomly transforming data within a dataset, the neural network has a lower chance of memorizing the training data and is required to adapt to different situations. In this submodule, the effect of data augmentation on the prediction accuracy of a ResNet-18 CNN was observed. When a purely augmented dataset is used to train a CNN, the ability for the network to accurately classify images decreases by roughly 20% relative to a network trained on an unmodified dataset. However, when the network is trained on a dataset containing both the original and augmented dataset, the network accuracy improves by about 5% compared to the original network. This increase is due to the random variations in the dataset that effectively increase the size of the overall dataset, meaning the network has a larger amount of data to train on [17]. As shown in this submodule, the ratio of original to augmented images allows for a balance between the network learning features within the dataset and improving the robustness of those learned features. However, the ratio of input images can also severely impact the ability for the network to learn features. In typical CNN training loops, a 50/50 split of original to augmented data is implemented but can be adjusted based on the final accuracy of the trained network.

### Submodule 3: segmentation

Semantic segmentation is the term that refers to the ability to generate a pixel-wise classification map of some data input. As the input data flow through a traditional CNN, features within the data become encoded and used to ultimately predict a single classification for a whole input. While this classification can be accurate, it lacks any spatial context within the input [32]. By utilizing both an encoding and decoding path within a U-Net CNN, the final output is the same size as the input, resulting in a map of classification values. In this submodule, a biomedical-related dataset of *in vivo* images is used to train the U-Net architecture to semantically segment the input image. Overall, this trained network is shown to produce relatively high accuracies (>85%).

### Submodule 4: regression

By combining regression and deep learning neural networks, networks can be trained to produce continuous values rather than discrete classifications. Although this submodule highlights the ability for a neural network to form a regression model, there are many other algorithms that can produce similar results. In these experiments, a tabular breast cancer dataset is used to predict the average radius of nuclei within the dataset. When simple linear regression, decision tree and random forest regression models are used with this dataset, the expected outcome is accurately predicted with a relatively high standard deviation in the MAE and RMSE (>0.01). When the regression neural network is trained, the average MAE and RMSE does not appreciably change (≈0.1), but the standard deviation is substantially lower (≤0.001).

## DISCUSSION

Deep learning is a powerful tool that leverages AI to predict outcomes based on input data. However, the broad use of deep learning in biomedical research is still not fully realized due to the complex nature of implementing a deep learning approach. The goal of this module is to provide an environment where biomedical researchers can learn the basics of deep learning and how to implement deep learning for biomedical research, while not requiring the costly computational equipment typically needed for deep learning tasks. In this module, Jupyter notebooks covering key deep learning topics were generated to provide examples of how to utilize classification, augmentation, segmentation and regression in biomedical research. To enable multiple routes of learning, code chunks as well as descriptive explanations for each code chunk are available within each submodule. Additionally, quizzes and code challenges are included to facilitate in-depth learning. All modules are available on the National Institutes of Health (NIH) National Institute of General Medical Sciences (NIGMS) Sandbox GitHub page, and instructions for cloning repositories are provided for each module. Each submodule is designed to be run within a cloud-computing environment and specifically the GCP. Any algorithm in these submodules can be easily modified by the researcher to use other datasets or run in another environment.

CNNs use filters made up of weights and biases to learn how to perform a task such as classification. The architecture and the weights and biases within the network dictate the ability for the network to accurately predict the classification of an input. Although the architecture of these networks can be somewhat arbitrary, there exist many pre-defined network architectures that can be used at a starting point for integrating deep learning with biomedical research. In submodule 1, the ResNet-18 architecture is shown to accurately classify histological images when trained from different initial conditions. When randomly initialized and trained on the PathMNIST dataset, a relatively high accuracy (≥90%) can be expected. Although the network learns key features about this dataset, this may not always be the case, especially for datasets with a large number of classes available. To remedy this issue, the learning rate of the network and the number of training epochs can be increased but results in a longer training time. Finally, when applying CNNs to a dataset, the choice of hyperparameter settings (i.e. training epochs, learning rate, etc.), may need to change based on the dataset and network architecture used. Furthermore, optimization of these hyperparameter settings must be carefully performed and may be required for robust CNN training. Although the image normalization and default training parameters used in the first submodule result in a well-performing neural network, these parameters may need to be adjusted based on the data that are inputted into the network. When a fine-tuning approach is used in submodule 1, the accuracy of the trained network drastically decreases if only the prediction layer is allowed to train. This decrease in accuracy is due to the intrinsic differences between the ImageNet dataset and the PathMNIST dataset, which are not able to be captured by just the final prediction layer [17]. When re-training the prediction layer is not sufficient, the whole network can be re-trained instead. By re-training the whole network, the initial weights and biases are not randomized compared to a ‘from scratch’ model, and the network is optimized for a new dataset, which may decrease the overall training time needed to develop a robust network.

Inherent biases within a dataset, as well as overfitting during the training process of a neural network, are issues that can greatly impact the robustness and generalizability of a trained neural network. The process of minimizing these issues is commonly known as regularization [7]. As shown in submodule 2, augmentation is a powerful regularization method that can improve the accuracy of a neural network by artificially increasing the number of images in a dataset through random cropping and horizontal/vertical flipping. When a fully randomized dataset is used, however, the ability for the neural network to accurately predict classes is greatly hindered. Alternatively, when a delicate balance of unmodified and augmented images are used, the accuracy of the network can improve relative to being trained on only an unmodified dataset. While the amount of unmodified and augmented images used in the combined dataset is arbitrary in submodule 2, the balance, as well as methods of augmentation, can be optimized via further testing for a specific biomedical dataset.

Depending on the application, a single output classification network may not be ideal. In cases where an input, such as an image, has multiple regions of interest with different classifications, a segmentation network can be trained to assign classification values to each base unit of the input. Alternatively, a regression network could also be trained if a continuous output is required. In this module, these concepts are explored and shown to provide relatively high prediction power for different types of datasets. Finally, although the key concepts for each of these networks are covered in this module, these methods can also be combined depending on the user requirements.

In summary, deep learning is an AI technique where a neural network is used to learn simple and complex features within a dataset to predict some outcome. This method is incredibly useful in biomedical image data analysis due to the ability to identify complex relationships within large datasets. In this module, users are encouraged to explore types of neural networks and deep learning methods that have been commonly used in biomedical-related datasets. From an education and teaching perspective, cloud computing platforms such as the GCP allow for trainees to be easily trained by removing the burden of required computational resources, such as multiple graphical processing units, and providing high-end resources at low cost (~$1 to run a full module). The NIGMS Sandbox, including this module, is publicly available on GitHub, so researchers from a wide range of public, private and academic affiliations are able to easily access this training module. While the modules within the NIGMS Sandbox are intended for cloud computing resources, the modules are free to be run on local machines once a module repository has been cloned. However, if this module is run locally, one must consider the computational resources available to the user. If large network architectures are too computationally expensive, one may consider using network architectures that contain less trainable parameters. While the initial scope of this module is to establish a beginning groundwork for incoming users to implement deep learning for biomedical research, the initial four submodules are relatively limited in their breadth. There are many avenues for module expansion in the future to generate a richer knowledge base. Some potential future directions include an introduction to other types of basic neural networks such as graph neural networks or recurrent neural networks and implementations of more complex networks such as generative adversarial networks or more cutting-edge algorithms such as those utilized in YOLOR [25].

**Key Points**

- Deep learning can yield interesting scientific outcomes, but proper training for biomedical researchers is required.
- Cloud computing platforms are useful in deep learning applications for reducing the computational burden of local hardware.
- Cloud computing platforms provide high-end resources to users at relatively low costs.

## Data Availability

The NIGMS sandbox is available at https://github.com/NIGMS/NIGMS-Sandbox, and this specific module is available at https://github.com/NIGMS/Biomedical-Imaging-Analysis-using-AI-ML-Approaches. Additionally, the Google Cloud storage bucket for submodule 3 is available at nigms-sandbox/nosi-uasm-alml/segmentation_data_small.

## Notes

**Alan E. Woessner** is a research associate at the University of Arkansas and the manager for the Imaging and Spectroscopy Core within the NIH-funded Arkansas Integrative Metabolic Research Center (AIMRC).

**Usman Anjum** is a research associate at University of Cincinnati, Ohio in the Computer Science Department. His research interests include machine learning and AI, specifically in natural language processing and application of AI in biomedical research.

**Hadi Salman** is a Computer Science PhD graduate from the University of Arkansas with expertise in computer vision.

**Jacob Lear** is a Computer Science PhD student at the University of Arkansas studying machine learning.

**Jeffrey T. Turner** is a data science specialist at Deloitte with a focus on deep learning, computer vision and machine learning.

**Ross Campbell** is a bioinformaticist at Deloitte with a background in biomarker development from multi-omics experiments. He has expertise in optimizing bioinformatics machine learning pipelines to run at scale in cloud environments.

**Laura Beaudry** is a program manager at Google. Her focus is on technical program management of engineering teams to ensure timely and impactful delivery of Google Cloud projects for federal agencies and organizations.

**Justin Zhan** is the Head of Department of Computer Science, College of Engineering and Applied Science, University of Cincinnati. His research interests include data science, artificial intelligence, blockchain technologies, biomedical informatics, information assurance and social computing.

**Lawrence Cornett** holds the rank of distinguished professor in the Department of Physiology and Cell Biology at the University of Arkansas for Medical Sciences. He is the director of the Arkansas IDeA Network of Biomedical Research Excellence (INBRE).

**Susan Gauch** is a professor of Electrical Engineering and Computer Science at the University of Arkansas whose research focuses on intelligent information retrieval and natural language processing.

**Kyle P. Quinn** is a professor of biomedical engineering at the University of Arkansas and director of the NIH-funded Arkansas Integrative Metabolic Research Center (AIMRC).
